# Preoperative frailty and chronic pain after cardiac surgery: a prospective observational study

**DOI:** 10.1186/s12871-022-01746-x

**Published:** 2022-07-01

**Authors:** Britta C. Arends, Leon Timmerman, Lisette M. Vernooij, Lisa Verwijmeren, Douwe H. Biesma, Eric P. A. van Dongen, Peter G. Noordzij, Heleen J Blussé van Oud-Alblas

**Affiliations:** 1 Department of Anesthesiology, Intensive Care, and Pain Medicine, St. Antonius Hospital, Koekoekslaan 1, 3430 EM Nieuwegein, The Netherlands; 2 Department of Anesthesiology, Intensive Care and Emergency Medicine, University Medical Center Utrecht, Utrecht, the Netherlands; 3 Department of Internal Medicine, Leiden University Medical Centre, Leiden, the Netherlands

**Keywords:** Chronic pain, Postoperative pain, Frailty, Elderly

## Abstract

**Background:**

Chronic pain after cardiac surgery, whether or not related to the operation, is common and has negative impact on health related quality of life (HRQL). Frailty is a risk factor for adverse surgical outcomes, but its relationship with chronic pain after cardiac surgery is unknown. This study aimed to address the association between frailty and chronic pain following cardiac surgery.

**Methods:**

This sub-study of the Anesthesia Geriatric Evaluation study included 518 patients ≥ 70 years undergoing elective cardiac surgery. Pain was evaluated with the Short-Form 36 questionnaire prior to and one year after surgery. Associations between chronic postoperative pain and frailty domains, including medication use, nutritional status, mobility, physical functioning, cognition, HRQL, living situation and educational level, were investigated with multivariable regression analysis.

**Results:**

Chronic pain one year after cardiac surgery was reported in 182 patients (35%). Medication use, living situation, mobility, gait speed, Nagi’s physical functioning and preoperative HRQL were frailty domains associated with chronic pain after surgery. For patients with chronic pain physical HRQL after one year was worse compared to patients without chronic pain (β –10.37, 99% CI –12.57 – –8.17).

**Conclusions:**

Preoperative polypharmacy, living alone, physical frailty and lower mental HRQL are associated with chronic pain following cardiac surgery. Chronic postoperative pain is related to worse physical HRQL one year after cardiac surgery. These findings may guide future preoperative interventions to reduce chronic pain and poor HRQL after cardiac surgery in older patients.

**Trial Registration:**

This trial has been registered before initiation under number NCT02535728 at clinicaltrials.gov.

**Supplementary Information:**

The online version contains supplementary material available at 10.1186/s12871-022-01746-x.

## Background

Chronic pain is a well-known complication after cardiac surgery and is reported by 18 to 35% of cardiac surgery patients in the Netherlands [1–3]. Especially in older patients, chronic pain, whether or not related to surgery, has a major impact on postoperative functional outcome, including health related quality of life (HRQL) [1–5]. Physical inactivity and reduced self-reliance due to chronic pain, have been associated with a greater vulnerability to stressors, social isolation, anxiety and depression [5–11]. Although preoperative anesthesiological assessment routinely includes risk stratification for cardiac or pulmonary complications, standardized screening for the risk to develop chronic pain is less common. Given the negative effects, it is essential that risk factors for chronic pain after surgery are identified in order to initiate preventive strategies.

Frailty is characterized by a limited resilience to surgical stress, and has been associated with poor postoperative outcomes [12–14]. In community-dwelling elderly, chronic pain has been related to frailty [13]. Frail patients have more pain, poorer daily functioning and less physical activity [13]. In the surgical population, frailty has been associated with chronic pain after major elective non-cardiac surgery [15]. Although frailty is considered an important risk factor for poor surgical outcomes, evidence of a relationship between frailty and chronic pain following cardiac surgery is lacking. Identification of a relation between specific preoperative frailty characteristics (domains) and postoperative chronic pain may guide interventions to improve surgical outcome. With an ageing population, the number of cardiac surgery procedures in older patients will rise in the upcoming years [4–6]. Optimizing preoperative circumstances in these patients is therefore essential to target analgesic interventions and preserve postoperative quality of life. We hypothesized that preoperative frailty domains are associated with chronic pain and worse HRQL one year after cardiac surgery in older patients.

This study therefore aimed to address whether specific frailty domains are associated with chronic pain following cardiac surgery in an older population. Additionally, the relationship of chronic pain to HRQL was evaluated..

## Methods

### Study design and population

This sub-study of the Anesthesia Geriatric Evaluation and quality of life after cardiac surgery (AGE) study analyzed patients included at St. Antonius Hospital, The Netherlands [16, 17]. The AGE study was a prospective observational cohort study in patients aged 70 years and older, that focused on the association between preoperative frailty with HRQL and disability after one year in elective cardiac surgery patients (i.e. coronary, valve, rhythm, aortic, or any combination of these procedures). The medical ethics committee approved the study protocol before patient recruitment (Medical Ethics Research Committees United (www.mec-u.nl), number R15.039). The study was first registered at clinicaltrials.gov under NCT02535728 at 31/08/2015. This manuscript adheres to the applicable STROBE guidelines. All participants provided written informed consent. Details on design and analyses of the AGE study have been previously reported [16].

### Clinical characteristics and data collection

After routine preoperative screening, an additional geriatric assessment was performed to assess physical, mental and social frailty in eleven domains. Physical frailty included the following domains: medication use, nutritional status using the Mini Nutritional Assessment [18] (MNA), mobility and gait speed using the Timed Get Up & Go test [19] (TGUG) and five-meter gait speed test [20] (5-MWT), daily physical functioning using Nagi’s scale [20] and a handgrip strength test [21]. Screening for mental frailty included cognition using the Minimal Mental State Examination [22] (MMSE) and self-rated mental and physical health with the Short-Form 36 questionnaire (SF-36) [23, 24]. To assess social frailty, we evaluated the living situation and educational level. Based on the multidimensionality of the frailty syndrome, a patient was considered ‘overall frail’ if a positive test for physical, mental and social frailty was present. An elaborate description of frailty tests and chosen cut-off values is described in additional file Table A1. Demographics and medical history were derived from the electronic health record, including health status, comorbidities, previous surgical procedures and/or laboratory tests. Data from the SF-36 was used to identify presence of preoperative pain (see ‘outcomes’ section below). Information on preoperative use of analgesics was retrospectively collected from electronic patient files and included acetaminophen, non-steroid anti-inflammatory drugs (NSAIDs), opioids and antidepressants. Opioids included intravenous and subcutaneous administered morphine, oxycodone hydrochloride controlled-release (Oxycontin), oxycodone hydrochloride immediate-release (Oxynorm) and tramadol. Antidepressants were selective serotonin reuptake inhibitors (SSRIs), tricyclic antidepressants (TCAs), pregabalin and amitriptyline. Polypharmacy and excessive polypharmacy were defined as ≥ 5 and < 10 prescriptions and ≥ 10 prescriptions, respectively.

### Perioperative analgesia

Perioperative care was routinely performed according to local standard operating procedures. For intraoperative analgesia a continuous infusion of remifentanil was initiated directly after induction of anesthesia and intermittent fentanyl doses were used at predetermined times (i.e. prior to incision of the skin, sternotomy, aorta cannulation and opening of the pericardium). The dose of remifentanil and fentanyl was determined at the discretion of the attending anesthesiologist, depending on patient characteristics and intraoperative vital parameters. All patients received a loading dose of 10 mg intravenous morphine 30 min before the anticipated end of surgery. Postoperative pain management at the intensive care unit (ICU) consisted of intravenous paracetamol (1 g every six hours) and a continuous infusion of morphine (1–2 mg/h) according to protocol. After ICU discharge a standardized postoperative pain protocol was started including Oxycontin 10 mg twice daily, Oxynorm 5 mg as needed (maximum 6 times a day) and paracetamol 1 g four times a day during the first and second day at the ward. On the third day at the ward opioids were reduced and Oxynorm 5 mg as needed was prescribed, together with paracetamol 1 g four times a day. From the fourth day onwards patients received paracetamol 1 g four times a day. Insufficient pain control was managed by consultation of the hospital acute pain service that advised on an individualized pain treatment plan. Patients that suffered chronic pain preoperatively continued their pain therapy, with the exception of NSAID use. Preoperative opioid use was taken into account when defining postoperative opioid dose.

### Outcomes

One year after cardiac surgery, study patients were invited by letter to complete and return the SF-36 questionnaire. One phone-call was used to remind non-responders. The primary outcome was chronic pain following cardiac surgery after 12 months. Data from the SF-36 questionnaires prior to and one year after surgery were used to determine chronic pain by the following question: ‘How much bodily pain did you have during the past 4 weeks?’ Answers were graded 1 to 6 and represented; ‘None’ (1), ‘Very Mild’ (2), ‘Mild’ (3), ‘Moderate’ (4), ‘Severe’ (5) and ‘Very Severe’ (6) [22, 23]. For this study, chronic pain was divided in three groups: ‘No pain’ (grade 1), ‘Mild pain’ (grade 2–3) and ‘Moderate to severe pain’ (grade 4–6). Chronic pain was defined as a reclassification into a higher grade of pain or no improvement of preexistent moderate to severe pain one year after cardiac surgery. The source or location of pain symptoms were not registered. Our secondary outcome was HRQL according to the SF-36 [23, 24]. HRQL was measured before, and at three and twelve months after surgery. Change in HRQL was expressed by a delta score between the preoperative measurement and at one year after surgery, consisting of eight sub scores (i.e. physical functioning, role functioning, role emotional, social functioning, bodily pain, mental health, vitality and general health). Sub scores ranged from 0 to 100 and were summarized into a mental HRQL and physical HRQL score, with positive values representing improvement. Death was scored as 0 points [16].

### Statistical analysis

Data are presented as frequencies and percentages (%) for categorical data and as median with interquartile range (IQR) or mean with standard deviation (SD) for continuous data, as appropriate. Normal distribution of the variables was assessed with visual inspection of the histograms and Q-Q plots. Differences between patients with and without chronic pain one year after surgery were compared using the Chi square test for dichotomous or categorical variables or the Mann–Whitney U test or Student’s T-test for continuous variables as appropriate. To investigate the association between each frailty domain and chronic pain one year after cardiac surgery, multivariable log-binomial regression analysis was performed to present effect estimates as risk ratios (RR) with accompanying 99% confidence interval (99% CI). To take multiple testing into account, we tested against a *p*-value of 0.01 and used a CI of 99%. Bonferroni adjustment was deemed inappropriate and too conservative as the different frailty domains are highly dependent on each other [17]. As chronic pain one year after cardiac surgery was relatively common, the rare disease assumption would not hold. This means that an odds ratio, would not approach the corresponding risk ratio, hampering the interpretation of our results for clinical practice [25]. All associations were adjusted for EuroSCORE II to take age, sex, comorbidities and weight of the procedure into account. Additionally, the association was adjusted for intraoperative use of remifentanil, preexisting chronic pain and use of internal mammary artery [1, 2, 26–30]. These confounders were a priori selected based on literature [1, 2, 26–30]. Next, change of HRQL in all eight sub scores prior to and one year after surgery was compared between patients with and without chronic pain using a Wilcoxon signed-rank test, for this univariate analysis *p*-values ≤ 0.01 were considered statistical significant. To investigate the association of chronic pain with HRQL after one year, multivariable linear regression models were conducted, where physical and mental HRQL measured at 12 months were used as the outcome. All associations were adjusted for EuroSCORE II, preexisting chronic pain, overall frailty and physical or mental HRQL measured prior to surgery. Estimates are expressed as linear regression coefficients (β) with accompanying 99% CI. To assess the robustness of our findings, sensitivity analysis were performed using the same analytical approaches. The first post-hoc analysis excluded all patients who died within 12 months of follow up. In the second post-hoc analysis, only patients with new or worse pain one year after surgery (i.e. reclassified into a higher grade of pain) were scored as chronic pain and patients with preexistent moderate to severe pain were excluded from this definition. As SF-36 data was missing for 11% of cases and could lead to potential bias, multiple imputation was conducted using the ‘mice’ library in R [31, 32]. Twenty data sets were created and the estimates and variances for each of the imputed datasets were pooled into an overall estimate using Rubin’s rule. The imputed dataset was used for final analyses. In order to obtain chronic pain categories after imputation, the mean frequencies of the specific answers to the SF-36 questionnaire at baseline and one year after surgery across the 20 imputation datasets were rounded to the nearest integer. An a priori sample size was not performed, as the sample size was based on the available data of the AGE study [16]. Data analysis was performed using R statistics (version 3.6.3, 2020).

## Results

### Study population

Overall, 518 patients were included in the analysis. Reasons for exclusion were withdrawal (*n* = 9) or cancellation of surgery (*n* = 17). Fifty-seven patients (11%) had one or more missing values (see additional file Table A2 for characteristics of patients with and without missing data). Prior to surgery, 91 patients (18%) were considered frail and chronic pain was reported by 331 patients (64%) of whom 77 (23%) were frail. Of all patients with preexisting chronic pain, 13% (44/331) used one analgesic and 7% (22/331) used two or more analgesics. Most common analgesics were acetaminophen (28/331, 9%), NSAIDs (18/331, 5%) and opioids (16/331, 5%). Patients with chronic pain prior to surgery more often used an antidepressant, 26/331 versus 4/187 (8% versus 2%), compared to patients without chronic pain prior to surgery (*p* = 0.01). Additional file Table A3 demonstrates the baseline characteristics for patients with and without chronic pain prior to surgery.

### Frailty and chronic pain after cardiac surgery

**Fig. 1:**
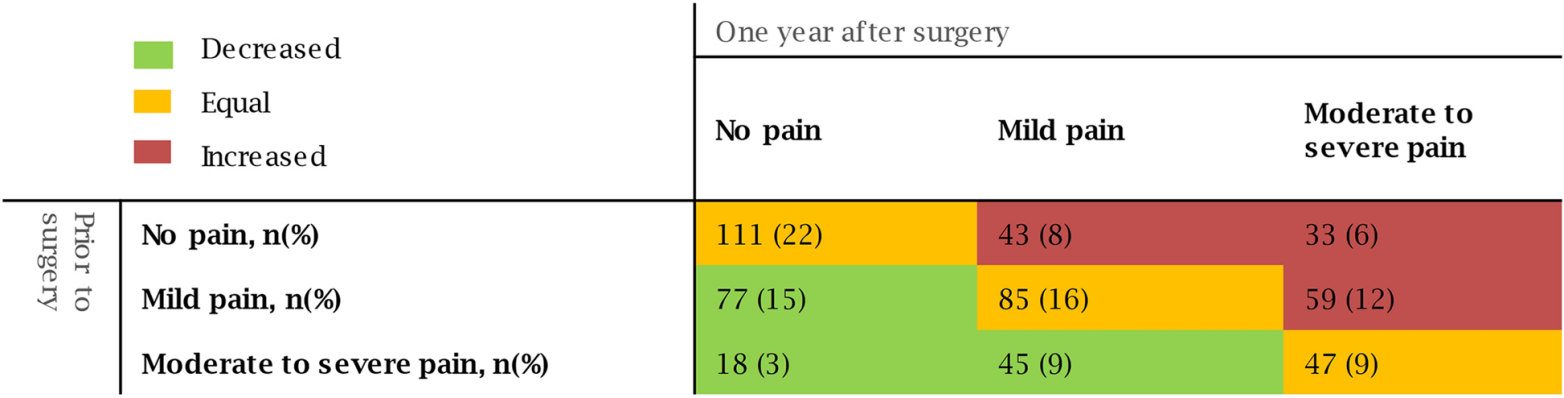
Pain intensity before and one year after cardiac surgery. *n*: Number

**Fig. 2:**
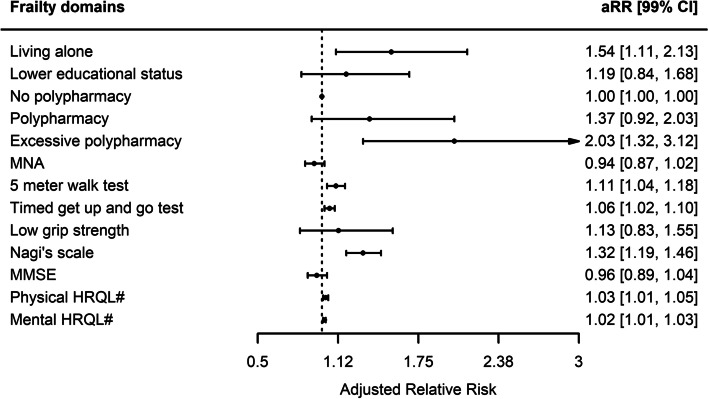
Adjusted relative risks for the development of chronic pain. *aRR* Adjusted relative risk, *CI* Confidence interval, *MMSE* Minimal mental state examination, *MNA* Mini-nutritional assessment, *HRQL* Health related quality of life. Polypharmacy was added as factor with polypharmacy defined as ≥ 5 and < 10 prescriptions and excessive polypharmacy defined as ≥ 10 prescriptions used. No polypharmacy was used as reference category. Log-binomial regression was used for statistical testing with correction for EuroSCORE II, intraoperative use of remifentanil, preexisting chronic pain and use of internal mammary artery. *P*-value ≤ 0.01 was considered statistically significant. #; per point decrease on physical and mental HRQL

One hundred forty patients (27%) reported improvement of pain, 243 (47%) had no or unchanged pain and 135 patients (26%) reported new or worse chronic pain one year after surgery (Fig. 1). According to our definition, chronic pain was present in 182 patients (35%), which included 47 patients with pre-existent moderate to severe pain that was not improved. Baseline characteristics according to chronic pain after cardiac surgery are presented in Table 1. Patients with chronic pain had higher EuroSCORE II at baseline, more often used opioids and had lower test results in the physical frailty domains. Patients preoperatively considered frail had a higher risk of developing postoperative chronic pain (aRR 1.58, 99% CI 1.08 – 2.30). Figure 2 demonstrates the association between each frailty domain and chronic pain one year after surgery. Medication use, living situation, mobility, gait speed, Nagi’s physical functioning and preoperative HRQL were associated with chronic pain after surgery. Patients with preoperative excessive polypharmacy, patients who were living alone and patient with lower mental HRQL had increased risks to develop chronic pain (aRR 2.03, 99% CI 1.32 – 3.12, 1.54, 99% CI 1.11 – 2.13, and aRR 1.02 99% CI 1.01 – 1.03 per point decrease on mental HRQL, respectively). Also, preoperative impaired physical functioning was associated with postoperative chronic pain (aRR 1.11, 99% CI 1.04 – 1.18 per second increase on 5-MWT, aRR 1.06, 99% CI 1.02 – 1.10 per second increase on TGUG, aRR 1.32, 99% CI 1.19 – 1.46 per point increase on Nagi’s scale and aRR 1.03 99% CI 1.01 – 1.05 per point decrease on physical HRQL). When patients with preexistent moderate to severe pain were excluded from the chronic pain definition in the post-hoc analysis, mobility and preoperative HRQL were no longer significantly associated (see figure in additional file Figure A1). Exclusion of patients who died within 12 months of follow-up did not change the associations (see figure in additional file Figure A2).

**Table 1:** Baseline patients characteristics

	**No chronic pain** (*n* = 336)	**Chronic pain** (*n* = 182)	***p*-value**
**Patient characteristics**			
Male sex	240 (71)	109 (60)	0.01
Age, years	74 (72 – 77)	75 (72 – 78)	0.09
BMI (kg∙m^−2^)	26.20 (24.20 – 28.70)	26.60 (24.42 – 30.10)	0.16
EuroSCORE II	1.69 (1.21 – 2.91)	2.16 (1.39 – 3.66)	0.001
Preoperative use of analgesics			
Acetaminophen	19 (6)	15 (8)	0.34
NSAIDs	12 (4)	11 (6)	0.28
Opioids	9 (3)	12 (7)	0.05
Antidepressants	14 (4)	16 (9)	0.05
Type of surgery			
Single CABG or maze	124 (37)	55 (30)	0.15
Single valve	91 (27)	53 (29)	0.70
Combined surgery	97 (29)	59 (32)	0.46
Aortic surgery	24 (7)	15 (8)	0.78
Duration of surgery, minutes	204 (163 – 250)	210 (168 – 258)	0.50
Remifentanyl (microgram)	2000 (1502 – 2000)	2000 (1079 – 2000)	0.004
Use of internal mammary artery	166 (49)	72 (40)	0.04
Length of stay in the ICU, days	1 (1 – 2)	1 (1 – 3)	0.04
Length of hospital stay, days	8 (7 – 12)	9 (7 – 15)	0.07
Complication (re-thoracotomy)	15 (5)	13 (7)	0.28
**Frailty domains**			
Living alone	57 (18)	53 (29)	< 0.01
Lower education	77 (23)	53 (29)	0.15
Polypharmacy	211 (63)	134 (74)	0.02
Excessive polypharmacy	42 (13)	46 (25)	< 0.001
MMSE, points	29 (28 – 30)	29 (27 – 30)	0.37
5 Meter walk test, seconds	4.6 (4.1 – 5.3)	4.9 (4.2 – 5.9)	< 0.001
Timed get up and go test, seconds	9.7 (8.5 – 11.2)	10.4 (8.7 – 12.8)	0.002
Low grip strength	118 (35)	71 (39)	0.45
Nagi’s scale, points	0 (0 – 1)	1 (0 – 2)	< 0.001
MNA, points	13 (12 – 14)	12.5 (11 – 14)	0.06
Mental HRQL, points	53.1 (44.1 – 58.0)	47.9 (37.7 – 54.0)	< 0.001
Physical HRQL, points	44.6 (35.7 – 52.0)	41.0 (32.1 – 49.3)	0.005

Continuous values as mean (± standard deviation) or median (1st to 3rd quartile), categorical values as frequency (%). *n* number, *BMI* Body mass index*, NSAIDs* Non-steroid anti-inflammatory drugs, *CABG* Coronary artery bypass grafting, *ICU* Intensive care unit, *MMSE* Minimal mental state examination, *MNA* Mini-nutritional assessment, *HRQL* Health related quality of life

### Chronic pain and quality of life at one year after surgery

**Fig. 3:**
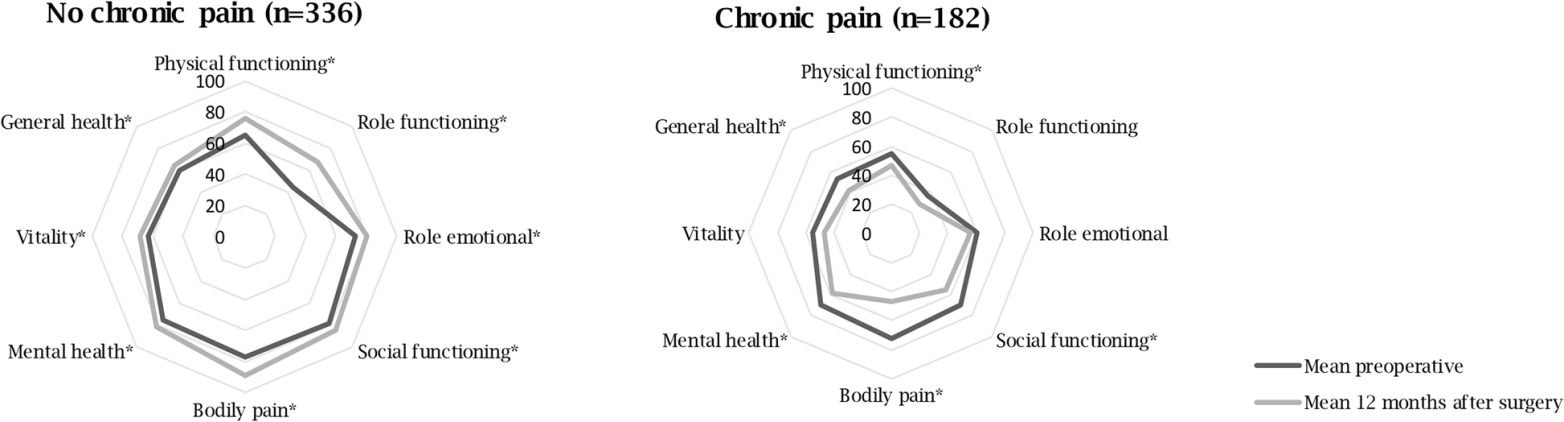
Change in health related quality of life in eight sub-scores.*p < *0.01, tested with Wilcoxon signed-rank test*

Figure 3 demonstrates the mean change HRQL in all eight sub scores prior to and one year after surgery in patients with and without chronic pain. Patients without chronic pain significantly improved in each sub score, where patients with chronic pain worsened. Multivariable linear regression analysis demonstrated that patients with chronic pain reported worse physical HRQL one year after surgery compared to patients without chronic pain (β –10.37, 99% CI –12.57 – –8.17). Chronic pain was not associated with mental HRQL after one year (β –0.83, 99% CI – 3.26 – 1.60). Results were similar after excluding patients who were deceased within one year after surgery and also after the exclusion of patients with preexistent moderate to severe pain from the chronic pain definition.

## Discussion

This study addressed the association between frailty domains and chronic pain following cardiac surgery in older patients. Additionally, the impact of chronic pain on HRQL in older patients was evaluated. One out of three elderly reported chronic pain after one year and frail patients had a higher risk of chronic pain following cardiac surgery. Frailty domains that were associated with chronic pain following surgery were medication use, living situation, mobility, gait speed, Nagi’s physical functioning and preoperative HRQL. In addition, we found that postoperative chronic pain was associated with worse physical HRQL one year after surgery.

This study confirmed that chronic pain is common in elderly cardiac surgery patients with a similar incidence reported in prior studies [1–3]. However, in our study the number of patients with postoperative chronic pain (*n* = 182) decreased compared to the number of patients with pain prior to surgery (*n* = 331), and 27% of patients (140/518) reported an improvement in pain. This might be explained by an improved functional capacity, decreased ischemic chest pain and lower levels of anxiety following cardiac surgery. Nevertheless, increased pain symptoms were common. Considering that chronic pain had a profound effect on HRQL, identification of risk factors for development of chronic pain, especially in the frail and vulnerable population, is important and might help to initiate preventive strategies. Several risk factors including younger age, psychological impairment, preexisting pain, internal mammary artery harvest, use of remifentanil and emergency surgery have been described to increase the risk of chronic pain following cardiac surgery [1, 2, 26–30]. In contrast to prior studies, these risk factors were not associated with higher risks of the development of chronic pain in our study. This is likely explained by differences between surgical cohorts. The AGE study consisted of a frailty-prone population undergoing a wide range of elective cardiac surgery procedures, and the mean age was higher than in other reports.

Frail patients had a higher risk of developing postoperative chronic pain following cardiac surgery. This association might be explained by impaired physical exertion as higher levels of activity have been described to reduce pain sensitivity by decreased pain facilitation and increased pain inhibition [33]. Conversely, preexistent pain is well-known to have an impact on physical activity [5, 6]. Furthermore, preexistent pain is known to be a risk factor for acute and chronic pain following surgery [1, 26, 27]. The question arises whether preexistent pain or impaired physical functioning (possibly due to pain or frailty) in these patients is the most relevant risk factor for the development of chronic pain. In our study, preexistent pain was not significantly associated with postoperative chronic pain. Also, in a post-hoc analysis, in which patients with preexistent moderate to severe pain were excluded from the chronic pain definition, our results did not change. Consistent with prior research, this underlines the association between impaired physical functioning and the development of chronic postoperative pain [33].

Besides impaired physical functioning, medication use, living situation and preoperative mental HRQL were associated with chronic postoperative pain. Polypharmacy is common in older patients, and might impede pain management for several reasons. Apart from age- and disease-related changes in physiology, disease-drug and drug-drug interactions might lead to a heterogeneity in response to medications and increased adverse drug effects. Frailty further increases this heterogeneity and thus frail elderly with polypharmacy may be more susceptible to adverse events [11]. Next to this, polypharmacy leads to medication non-adherence, leading to a suboptimal effect of prescribed analgesic therapy [34]. In our study, patients with excessive polypharmacy had a twofold risk to develop chronic pain. Finally, patients living alone are prone to social isolation, which contributes to feelings of depression or anxiety, and a more intense experience of pain [11, 26, 35].

Gender has been described to interact with multiple preoperative factors as well as cardiac surgery outcomes [36]. Female gender has been positively associated with preoperative frailty, psychological disease and dementia in cardiac surgery patients [36]. The results of our study confirm the well-investigated relationship between female gender and chronic pain [26, 27]. When defining interventions to improve outcome following cardiac surgery based on preoperative risk stratification, gender-related disparities should be taken into consideration.

Our study confirmed the existing relationship between chronic pain and HRQL. In general, polypharmacy, physical inactivity, reduced self-reliance and social isolation leads to an increase in health consumption, pain and poor HRQL [11, 27, 28, 37, 38]. In addition, several studies found that pain adversely affects recovery and HRQL, and that the impact correlated with the severity of pain [27, 28, 37]. In patients with chronic pain in our study, mental and physical HRQL were lower prior to surgery and physical HRQL was worse one year after surgery compared to patients without chronic pain (*p* < 0.001). Understanding factors that are related to HRQL in older people can be used to preoperatively accommodate patients’ needs and preserve quality of life.

Risk stratification should lead to individualized evaluation and preparation for surgery. However, evidence for pre-habilitation is limited for cardiac surgery patients. However, preoperative exercise has been demonstrated to improve functional recovery [39]. Optimization of treatment expectations by a simple psychological intervention have shown to improve disability [40]. Currently, trials on pre-habilitation are being performed in cardiac surgery patients, but the results have to be awaited [41, 42].

Comprehensive evaluation of pharmacotherapy should be part of each preoperative assessment, but deserves additional attention of, for example, a pharmacist or geriatrician in patients with polypharmacy. Patients suffering from chronic pain preoperatively should receive an individualized perioperative pain management plan, depending on their preoperative situation. Within this plan, additional pharmacotherapy, locoregional anesthesia and/or non-pharmacological interventions including may be considered to treat acute postoperative pain and prevent the increase of chronic pain symptoms following cardiac surgery.

The following limitations should be considered. First, pain was determined by a health survey that was not specifically designed to assess pain or pain interference. This study population reported pain within the last 4 weeks at 12 months follow up after surgery, and defined it as chronic pain [43]. Unfortunately, differentiation between thoracic pain, wound pain, chest pain, pain due to the surgical procedure or other pain, and type of pain (i.e., neuropathic, musculoskeletal, inflammatory, or mechanical pain) was not possible [43]. Second, a single point estimate was used for the incidence of chronic pain which may have resulted in an underestimation. Besides, the ageing process may account for differences in pain signaling and perception, causing an inconsistence and variety in pain measurements. More specific, with ageing a loss in structure and function of peripheral nerves occurs [10, 44]. Due to a decrease in the spread and magnitude of brain activation in response to pain in elderly, pain thresholds might be higher [10, 44]. On the other hand, endogenous pain modulation in elderly shows age-related impairment [10, 44]. In particular, inhibitory systems were reported to be affected, resulting in lower capacities to modulate pain. This inadequacy to modulate pain leads to an increased risk for chronic pain. Finally, we did not register age-dependent conditions such as arthrosis and neurological conditions which may be related to frailty as well as to chronic pain. Further analysis of the reason for frailty may improve the prediction of chronic postoperative pain. Future research to explore these current findings should determine pain using patient diaries with validated pain assessments.

## Conclusions

Postsurgical chronic pain is common in elderly cardiac surgery patients. Preoperative polypharmacy, living alone, physical frailty, living alone and lower mental HRQL were positively associated to chronic pain following cardiac surgery. Secondly, chronic pain was associated with worse physical HRQL following cardiac surgery. The results of our study advocate that early identification of these factors may be used to identify older patients at risk for chronic pain after cardiac surgery.

## Supplementary Information

**Additional file 1:** **Figure A1. **Adjusted relative risks for the development of new or worse chronic pain, sensitivity analysis (*n*=135). *aRR* Adjusted relative risk, *CI* Confidence interval, *MMSE* Minimal mental state examination, *MNA* Mini-nutritional assessment, *HRQL* Health related quality of life. Polypharmacy was added as factor with polypharmacy defined as ≥5 and <10 prescriptions and excessive polypharmacy defined as ≥10 prescriptions used. No polypharmacy was used as reference category. Log-binomial regression was used for statistical testing with correction for EuroSCORE II, intraoperative use of remifentanil, preexisting chronic pain and use of internal mammary artery. *P*-value ≤0.01 was considered statistically significant. #; per point decrease on physical and mental HRQL.

**Additional file 2:** **Figure A2. **Adjusted relative risks for the development of chronic pain, sensitivity analysis without deceased patients (*n*=488). *aRR* Adjusted relative risk, *CI* Confidence interval, *MMSE* Minimal mental state examination, *MNA* Mini-nutritional assessment, *HRQL* Health related quality of life. Polypharmacy was added as factor with polypharmacy defined as ≥5 and <10 prescriptions and excessive polypharmacy defined as ≥10 prescriptions used. No polypharmacy was used as reference category. Log-binomial regression was used for statistical testing with correction for EuroSCORE II, intraoperative use of remifentanil, preexisting chronic pain and use of internal mammary artery. *P*-value ≤0.01 was considered statistically significant. #; per point decrease on physical and mental HRQL.

**Additional file 3:** **Table A1. **Description of frailty domains.

**Additional file 4:** **Table A2. **Baseline for patients with and without missing data.

**Additional file 5:** **Table A3. **Baseline for patients with and without chronic pain prior to surgery (*n* = 518). 

## Data Availability

The datasets used and/or analyzed during the current study are available from the corresponding author on reasonable request.

## Abbreviations

HRQL: Health related quality of life
aRR: Adjusted relative risk
95% CI: 95% Confidence interval
AGE: Anesthesia Geriatric Evaluation
MNA: Mini Nutritional Assessment
TGUG: Timed Get Up & Go test
5-MWT: Five-meter gait speed test
MMSE: Minimal Mental State Examination
SF-36: Short-Form 36 questionnaire
NSAIDs: Non-steroid anti-inflammatory drugs
Oxycontin: Oxycodone hydrochloride controlled-release
Oxynorm: Oxycodone hydrochloride immediate-release
SSRIs: Selective serotonin reuptake inhibitors
TCAs: Tricyclic antidepressants
ICU: Intensive care unit
IQR: Interquartile range
SD: Standard deviation
RR: Risk ratios
AIC: Akaike Information Criterion
REML: Restricted maximum likelihood estimation

## References

[1] Van Gulik L, Janssen LI, Ahlers SJGM, Bruins P, Driessen AHG, Van Boven WJ (2011). Risk factors for chronic thoracic pain after cardiac surgery via sternotomy. Eur J Cardio-thoracic Surg.

[2] Van Gulik L, Ahlers SJGM, Van De Garde EMW, Bruins P, Van Boven WJ, Tibboel D (2012). Remifentanil during cardiac surgery is associated with chronic thoracic pain 1 yr after sternotomy. Br J Anaesth.

[3] De Hoogd S, Ahlers SJGM, van Dongen EPA, Van De Garde EMW, Daeter EJ, Dahan A (2018). Randomized Controlled Trial on the Influence of Intraoperative Remifentanil versus Fentanyl on Acute and Chronic Pain after Cardiac Surgery. Pain Pract.

[4] Nicolini F, Agostinelli A, Vezzani A, Manca T, Benassi F, Molardi A (2014). The evolution of cardiovascular surgery in elderly patient: A review of current options and outcomes. Biomed Res Int..

[5] Saraiva MD, Suzuki GS, Lin SM, de Andrade DC, Jacob-Filho W, Suemoto CK (2018). Persistent pain is a risk factor for frailty: A systematic review and meta-analysis from prospective longitudinal studies. Age Ageing.

[6] Scandroglio MM, Finco G, Pieri M, Ascari R, Calabrò MG, Tadeo D (2015). Cardiac surgery in 260 octogenarians: A case series. BMC Anesthesiol.

[7] Achterberg WP, De Ruiter CM, De Weerd CMEE, Geels P, Horikx A, Verduijn MM (2012). [Multidisciplinary guideline ’Recognition and treatment of chronic pain in vulnerable elderly people’] [Dutch] Multidisciplinaire richtlijn “Herkenning en behandeling van chronische pijn bij kwetsbare ouderen”. Ned Tijdschr Geneeskd.

[8] Van Kleef M, Geurts JW (2012). Useful guideline for treatment of pain in vulnerable elderly people. Ned Tijdschr Geneeskd.

[9] Thomas E, Peat G, Harris L, Wilkie R, Croft PR (2004). The prevalence of pain and pain interference in a general population of older adults: Cross-sectional findings from the North Staffordshire Osteoarthritis Project (NorStOP). Pain.

[10] Paladini A, Fusco M, Coaccioli S, Skaper SD, Varrassi G (2015). Chronic pain in the elderly: The case for new therapeutic strategies. Pain Physician.

[11] McLachlan AJ, Bath S, Naganathan V, Hilmer SN, Le Couteur DG, Gibson SJ (2011). Clinical pharmacology of analgesic medicines in older people: Impact of frailty and cognitive impairment. Br J Clin Pharmacol.

[12] Lin HS, Watts JN, Peel NM, Hubbard RE (2016). Frailty and post-operative outcomes in older surgical patients: A systematic review. BMC Geriatr.

[13] Hirase T, Kataoka H, Nakano J, Inokuchi S, Sakamoto J, Okita M (2018). Impact of frailty on chronic pain, activities of daily living and physical activity in community-dwelling older adults: A cross-sectional study. Geriatr Gerontol Int.

[14] Clegg A, Young J, Iliffe S, Rikkert MO, Rockwood K (2013). Frailty in elderly people. Lancet.

[15] Esses GJ, Liu X, Lin HM, Khelemsky Y, Deiner S (2019). Preoperative frailty and its association with postsurgical pain in an older patient cohort. Reg Anesth Pain Med.

[16] Verwijmeren L, Peelen LM, van Klei WA, Daeter EJ, van Dongen EPA, Noordzij PG (2020). Anaesthesia geriatric evaluation to guide patient selection for preoperative multidisciplinary team care in cardiac surgery. Br J Anaesth.

[17] Arends BC (2022). Blussé van Oud-Alblas HJ, Vernooij LM, Verwijmeren L, Biesma DH, Knibbe CA, Noordzij PG, van Dongen EPA, The association of polypharmacy with functional decline in elderly patients undergoing cardiac surgery. Br J Clin Pharmacol.

[18] Rubenstein LZ, Harker JO, Salvà A, Guigoz Y, Vellas B (2001). Screening for undernutrition in geriatric practice: Developing the Short-Form Mini-Nutritional Assessment (MNA-SF). J Gerontol - Ser A Biol Sci Med Sci.

[19] Huisman MG, Van Leeuwen BL, Ugolini G, Montroni I, Spiliotis J, Stabilini C (2014). “Timed Up & Go”: A screening tool for predicting 30-day morbidity in onco-geriatric surgical patients? A multicenter cohort study. PLoS ONE.

[20] Afilalo J, Mottillo S, Eisenberg MJ, Alexander KP, Noiseux N, Perrault LP (2012). Addition of frailty and disability to cardiac surgery risk scores identifies elderly patients at high risk of mortality or major morbidity. Circ Cardiovasc Qual Outcomes.

[21] Sultan P, Hamilton MA, Ackland GL (2012). Preoperative muscle weakness as defined by handgrip strength and postoperative outcomes: A systematic review. BMC Anesthesiol.

[22] Folstein MF, Folstein SE, McHugh PR (1975). “Mini-mental state”. A practical method for grading the cognitive state of patients for the clinician. J Psychiatr Res.

[23] Ware JE, Sherbourne CD (1992). The MOS 36-item short-form health survey (Sf-36): I. conceptual framework and item selection. Med Care.

[24] Aaronson NK, Muller M, Cohen PDA, Essink ML, Fekkes M, Sanderman R (1998). Translation, validation, and norming of the Dutch language version of the SF-36 Health Survey in community and chronic disease populations. J Clin Epidemiol.

[25] Knol MJ, Le Cessie S, Algra A, Vandenbroucke JP, Groenwold RHH (2012). Overestimation of risk ratios by odds ratios in trials and cohort studies: Alternatives to logistic regression. CMAJ.

[26] Choinière M, Watt-Watson J, Victor JC, Baskett RJF, Bussières JS, Carrier M (2014). Prevalence of and risk factors for persistent postoperative nonanginal pain after cardiac surgery: A 2-year prospective multicentre study. CMAJ.

[27] Guimarães-Pereira L, Farinha F, Azevedo L, Abelha F, Castro-Lopes J (2016). Persistent Postoperative Pain after Cardiac Surgery: Incidence, Characterization, Associated Factors and its impact in Quality of Life. Eur J Pain.

[28] Gjeilo KH, Stenseth R, Wahba A, Lydersen S, Klepstad P (2017). Chronic postsurgical pain in patients 5 years after cardiac surgery: A prospective cohort study. Eur J Pain.

[29] Cohen AJ, Moore P, Jones C, Miner TJ, Carter WR, Zurcher RP (1993). Effect of internal mammary harvest on postoperative pain and pulmonary function. Ann Thorac Surg.

[30] Meyerson J, Thelin S, Gordh T, Karlsten R (2001). The incidence of chronic post-sternotomy pain after cardiac surgery - A prospective study. Acta Anaesthesiol Scand.

[31] van Buuren S, Groothuis-Oudshoorn K (2011). mice: Multivariate imputation by chained equations in R. J Stat Softw.

[32] Donders ART, van der Heijden GJMG, Stijnen T, Moons KGM (2006). Review: A gentle introduction to imputation of missing values. J Clin Epidemiol.

[33] Law LF, Sluka KA (2017). How does physical activity modulate pain?. Pain.

[34] Markotic F, Cerni Obrdalj E, Zalihic A, Pehar R, Hadziosmanovic Z, Pivic G (2013). Adherence to pharmacological treatment of chronic nonmalignant pain in individuals aged 65 and older. Pain Med.

[35] da Costa MAC, Trentini CA, Schafranski MD, Pipino O, Gomes RZ, Reis ESDS (2015). Factors associated with the development of chronic post-sternotomy pain: A case-control study. Brazilian J Cardiovasc Surg.

[36] Johnston A, Mesana TG, Lee DS, Eddeen AB, Sun LY (2019). Sex Differences in Long-Term Survival After Major Cardiac Surgery: A Population-Based Cohort Study. J Am Heart Assoc.

[37] Kalso E, Mennander S, Tasmuth T, Nilsson E (2001). Chronic post-sternotomy pain. Acta Anaesthesiol Scand.

[38] McIsaac DI, Wong CA, Bryson GL, Van Walraven C (2018). Association of Polypharmacy with Survival, Complications, and Healthcare Resource Use after Elective Noncardiac Surgery: A Population-based Cohort Study. Anesthesiology.

[39] Zheng YT, Zhang JX (2020). Preoperative exercise and recovery after cardiac surgery: a meta-analysis. BMC Cardiovasc Disord.

[40] Rief W, Shedden-Mora MC, Laferton JA, Auer C, Petrie KJ, Salzmann S, Schedlowski M, Moosdorf R (2017). Preoperative optimization of patient expectations improves long-term outcome in heart surgery patients: results of the randomized controlled PSY-HEART trial. BMC Med.

[41] Yau DKW, Wong MKH, Wong WT, Gin T, Underwood MJ, Joynt GM (2019). PREhabilitation for improving QUality of recovery after ELective cardiac surgery (PREQUEL) study: protocol of a randomised controlled trial. BMJ Open.

[42] Stammers AN, Kehler DS, Afilalo J, Avery LJ, Bagshaw SM, Grocott HP (2015). Protocol for the PREHAB study-Pre-operative Rehabilitation for reduction of Hospitalization After coronary Bypass and valvular surgery: a randomised controlled trial. BMJ Open.

[43] Treede RD, Rief W, Barke A, Aziz Q, Bennet MI, Benoliel R (2015). A classification of chronic pain for ICD-11. Pain.

[44] Gulur P, Nelli A (2019). Persistent postoperative pain: Mechanisms and modulators. Curr Opin Anaesthesiol.

