# Supplementary material for: Preoperative frailty and chronic pain after cardiac surgery: a prospective observational study
Source: BMC Anesthesiol. 2022 Jul 1;22:201. doi: 10.1186/s12871-022-01746-x (PMC9248159; doi:10.1186/s12871-022-01746-x)
Supplement: Supplementary file 1 — Additional file 1: Figure A1. Adjusted relative risks for the development of new or worse chronic pain, sensitivity analysis (n=135). aRR Adjusted relative risk, CI Confidence interval, MMSE Minimal mental state examination, MNA Mini-nutritional assessment, HRQL Health related quality of life. Polypharmacy was added as factor with polypharmacy defined as ≥5 and <10 prescriptions and excessive polypharmacy defined as ≥10 prescriptions used. No polypharmacy was used as reference category. Log-binomial regression was used for statistical testing with correction for EuroSCORE II, intraoperative use of remifentanil, preexisting chronic pain and use of internal mammary artery. P-value ≤0.01 was considered statistically significant. #; per point decrease on physical and mental HRQL. [file 12871_2022_1746_MOESM1_ESM.docx]

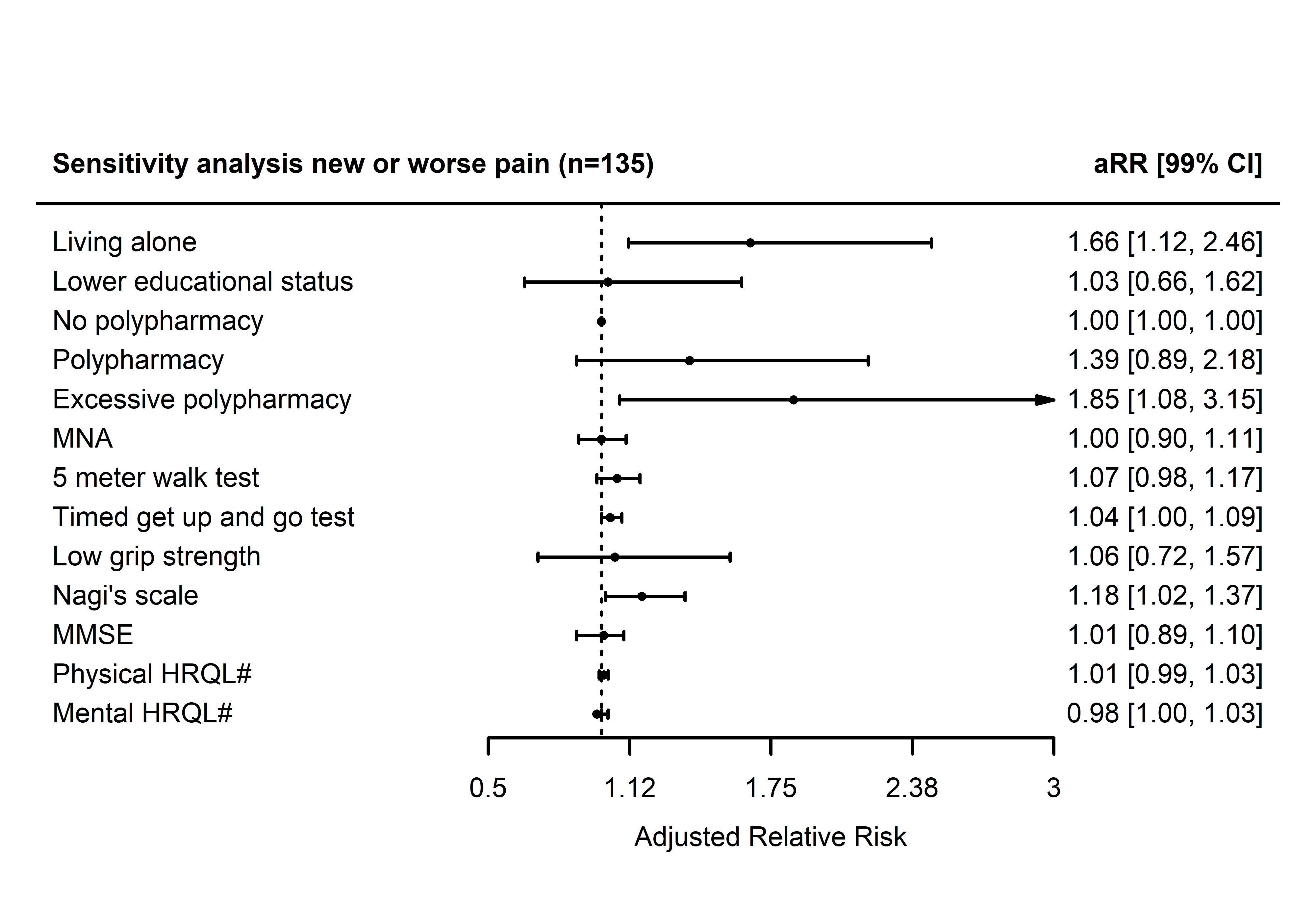


**Figure A1. Adjusted relative risks for the development of new or worse chronic pain, sensitivity analysis (n=135).**

aRR: adjusted relative risk; CI: confidence interval; MMSE: minimal mental state examination; MNA: mini-nutritional assessment; HRQL: health related quality of life. Polypharmacy was added as factor with polypharmacy defined as ≥5 and <10 prescriptions and excessive polypharmacy defined as ≥10 prescriptions used. No polypharmacy was used as reference category. Log-binomial regression was used for statistical testing with correction for EuroSCORE II, intraoperative use of remifentanil, preexisting chronic pain and use of internal mammary artery. P-value ≤0.01 was considered statistically significant. #; per point decrease on physical and mental HRQL.
