# Supplementary material for: Preoperative frailty and chronic pain after cardiac surgery: a prospective observational study
Source: BMC Anesthesiol. 2022 Jul 1;22:201. doi: 10.1186/s12871-022-01746-x (PMC9248159; doi:10.1186/s12871-022-01746-x)
Supplement: Supplementary file 3 — Additional file 3: Table A1. Description of frailty domains. [file 12871_2022_1746_MOESM3_ESM.docx]

**Table A1. Description of frailty domains.**

BMI: body mass index.

| Frailty domain | Test | Method of assessment | Cut off value |
| --- | --- | --- | --- |
| Nutritional status | Mini nutritional assessment | Six item questionnaire on weight loss, eating, BMI, and psychological status | A score ≤11 out of 14 identified patients at risk for malnutrition^17^ |
| Gait speed | 5-meter walk test | Patients were instructed to walk at their normal pace with walking aids if needed for five meters. Time between first footfall after the starting line and first footfall after the five meter line was recorded | Impaired gait speed was defined as ≥6 seconds  or inability to perform the test^19^ |
|  | Timed get up and go test | Time was recorded between standing up from a seated position in a chair, walk for three meters with walking aids if needed and return to a seated position | Impaired gait speed was defined as ≥10 seconds  or inability to perform the test^18^ |
| Polypharmacy | Number of prescriptions | Assessment by hospital pharmacy services | ≥5 and <10,  ≥10 prescriptions (excessive)^40^ |
| Daily functioning | Nagi’s scale of physical disability | Seven item questionnaire on lifting heavy objects, kneeling, raising arms above the head, walking one flight of stairs, and walking 1.5 kilometers | A score ≥3 implied impairments^19^ |
| Handgrip strength | Hydraulic handheld dynamometer | Best result of three consecutive tests to squeeze dynamometer with lower arm unsupported and in 90⁰ angle. | According to age and sex^20^ |
| Cognition | Mini Mental State Examination | Eleven item questionnaire on orientation in time and place, short term memory, attention, and following verbal and written commands. | A score of ≤25 out of 30 was considered as mildly impaired cognition^21^ |
| Health related quality of life | Medical Outcomes Study Short Form 36 | 36 item self-assessed questionnaire on physical and mental well-being generating two scores representing mental and physical health related quality of life | A deviation of >1 standard deviation from the population mean was considered impaired^22,23^ |
| Living situation | Interview | Impaired when a patient was living without a partner or family |  |
| Educational status | Interview | Impaired when no education beyond secondary education was followed |  |
