# Supplementary material for: Preoperative frailty and chronic pain after cardiac surgery: a prospective observational study
Source: BMC Anesthesiol. 2022 Jul 1;22:201. doi: 10.1186/s12871-022-01746-x (PMC9248159; doi:10.1186/s12871-022-01746-x)
Supplement: Supplementary file 4 — Additional file 4: Table A2. Baseline for patients with and without missing data. [file 12871_2022_1746_MOESM4_ESM.docx]

**Table A2. Baseline for patients with and without missing data.**

|  | **With missing data** (n = 57) | **Without missing data** (n = 461) | **p-value** | **Missing** |
| --- | --- | --- | --- | --- |
| **Patient characteristics** | | | |  |
| Male sex | 41 (72) | 308 (69) | 0.53 | 0 |
| Age, years | 75 (73 – 77) | 74 (72 – 77) | 0.14 | 0 |
| BMI (kg∙m^-2^) | 25.90 (24.60 – 28.70) | 26.30 (24.20 – 29.10) | 0.62 | 0 |
| EuroSCORE II | 2.33 (2.40 – 3.62) | 1.80 (1.24 – 3.11) | 0.03 | 0 |
| Preoperative use of analgesics  Acetaminophen  NSAIDs  Opioids  Antidepressants | 3 (5) 3 (5) 2 (4) 2 (4) | 31 (7) 20 (4) 19 (4) 28 (6) | 0.89 1 1 0.63 | 0 |
| Type of surgery  Single CABG or maze  Single valve  Combined surgery  Aortic surgery | 17 (30) 8 (14) 24 (42) 8 (14) | 162 (35) 136 (30) 132 (28) 31 (7) | 0.52 0.02 0.05 0.09 | 0 |
| Duration of surgery, minutes | 214 (178 – 318) | 206 (162 – 250) | 0.04 | 0 |
| Remifentanyl (microgram) | 1642 (1010 – 2000) | 2000 (1458 – 2000) | 0.001 | 17 |
| Use of internal mammary artery | 27 (47) | 211 (46) | 0.93 | 0 |
| Length of stay in the ICU, days | 2 (1 – 4) | 1 (1 – 2) | 0.001 | 0 |
| Length of hospital stay, days | 10 (7 – 19) | 8 (7 – 13) | 0.13 | 6 |
| Complication (re-thoracotomy) | 8 (14) | 20 (4) | 0.01 | 0 |
| **Frailty domains** | | | |  |
| Living alone | 16 (28) | 94 (20) | 0.24 | 0 |
| Lower education | 18 (32) | 112 (24) | 0.30 | 12 |
| Polypharmacy | 39 (68) | 306 (66) | 0.87 | 0 |
| Excessive polypharmacy | 12 (21) | 76 (16) | 0.50 | 0 |
| MMSE, points | 29 (27 - 30) | 29 (28 - 30) | 0.14 | 5 |
| 5 Meter walk test, seconds | 4.8 (4.1 - 5.5) | 4.7 (4.1 - 5.1) | 0.61 | 6 |
| Timed get up and go test, seconds | 9.8 (8.5 - 12.0) | 9.8 (8.6 - 11.6) | 0.73 | 6 |
| Low grip strength | 24 (42) | 165 (36) | 0.44 | 1 |
| Nagi’s scale, points | 0.0 (0.0 - 2.0) | 0.0 (0.0 - 1.0) | 0.66 | 2 |
| MNA, points | 13 (11 - 14) | 13 (12 - 14) | 0.51 | 0 |
| Mental HRQL, points | 52.4 (41.6 - 56.1) | 51.6 (42.1 - 57.4) | 0.76 | 7 |
| Physical HRQL, points | 45.2 (37.0 - 52.2) | 42.6 (33.7 - 51.0) | 0.12 | 7 |

Continuous values as mean (± standard deviation) or median (1st to 3rd quartile), categorical values as frequency (%). n: number; BMI: body mass index; NSAIDs: non-steroid anti-inflammatory drugs; CABG: coronary artery bypass grafting; ICU: intensive care unit; MMSE: minimal mental state examination; MNA: mini-nutritional assessment; HRQL: health related quality of life.
