# Supplementary material for: Preoperative frailty and chronic pain after cardiac surgery: a prospective observational study
Source: BMC Anesthesiol. 2022 Jul 1;22:201. doi: 10.1186/s12871-022-01746-x (PMC9248159; doi:10.1186/s12871-022-01746-x)
Supplement: Supplementary file 5 — Additional file 5: Table A3. Baseline for patients with and without chronic pain prior to surgery (n = 518). [file 12871_2022_1746_MOESM5_ESM.docx]

**Table A3. Baseline for patients with and without chronic pain prior to surgery (n = 518).**

|  | **No chronic pain prior to surgery** (n = 187) | **Chronic pain prior to surgery**  (n = 331) | **p-value** |
| --- | --- | --- | --- |
| **Patient characteristics** | | | |
| Male sex | 135 (72) | 214 (65) | 0.10 |
| Age, years | 74 (72 – 77) | 75 (72 – 77) | 0.85 |
| BMI (kg∙m^-2^) | 25.80 (23.75 – 27.90) | 26.90 (24.50 – 29.75) | 0.001 |
| EuroSCORE II | 1.88 (1.22 – 3.21) | 1.81 (1.25 – 3.28) | 0.92 |
| Preoperative use of analgesics  Acetaminophen  NSAIDs  Opioids  Antidepressants | 6 (3) 6 (3) 5 (3) 4 (2) | 28 (9) 17 (5) 16 (5) 26 (8) | 0.03 0.42 0.33 0.01 |
| Type of surgery  Single CABG  Single valve  Combined surgery  Aortic surgery | 45 (24) 60 (32) 63 (34) 19 (10) | 134 (41) 84 (25) 93 (28) 20 (6) | < 0.001 0.13 0.22 0.13 |
| Duration of surgery, minutes | 212 (160.5 – 262.5) | 205 (166.5 – 249) | 0.35 |
| Remifentanyl (microgram) | 2000 (1254.50 – 2000) | 2000 (1420 – 2000) | 0.16 |
| Use of internal mammary artery | 75 (40) | 163 (49) | 0.06 |
| Length of stay in the ICU, days | 1 1 – 2.5) | 1 (1 – 2) | 0.19 |
| Length of hospital stay, days | 9 (7 – 13) | 9 (7 – 13) | 0.91 |
| Complication (re-thoracotomy) | 8 (4) | 20 (6) | 0.52 |
| **Frailty domains** | | | |
| Living alone | 30 (16) | 80 (21) | 0.04 |
| Lower education | 39 (21) | 91 (27) | 0.12 |
| Polypharmacy | 103 (55) | 242 (73) | < 0.001 |
| Excessive polypharmacy | 22 (12) | 66 (20) | 0.02 |
| MMSE, points | 29 (28 - 30) | 29 (27 - 30) | 0.15 |
| 5 Meter walk test, seconds | 4.5 (4.0 - 5.1) | 4.8 (4.2 - 5.7) | < 0.001 |
| Timed get up and go test, seconds | 9.5 (8.3 - 11) | 10.1 (8.7 - 12.1) | < 0.001 |
| Low grip strength | 61 (33) | 128 (39) | 0.19 |
| Nagi’s scale, points | 0 ( 0 - 1) | 1 (0 - 2) | < 0.001 |
| MNA, points | 13 (12 - 14) | 13 ( 12 - 14) | 0.16 |
| Mental HRQL, points | 53.1 (44.0 - 57.6) | 51.3 (40.3 - 57.0) | 0.16 |
| Physical HRQL, points | 51.3 (45.2 - 54.8) | 37.6 (30.5 - 45.7) | < 0.001 |

Continuous values as mean (± standard deviation) or median (1^st^ to 3^rd^ quartile), categorical values as frequency (%). n: number; BMI: body mass index; NSAIDs: non-steroid anti-inflammatory drugs; CABG: coronary artery bypass grafting; ICU: intensive care unit; MMSE: minimal mental state examination; MNA: mini-nutritional assessment; HRQL: health related quality of life.
